# Delineation of Tumor Migration Paths by Using a Bayesian Biogeographic Approach

**DOI:** 10.3390/cancers11121880

**Published:** 2019-11-27

**Authors:** Antonia Chroni, Tracy Vu, Sayaka Miura, Sudhir Kumar

**Affiliations:** 1 Institute for Genomics and Evolutionary Medicine, Temple University, Philadelphia, PA 19122, USA; tug86468@temple.edu (T.V.); sayaka.miura@temple.edu (S.M.); s.kumar@temple.edu (S.K.); 2 Department of Biology, Temple University, Philadelphia, PA 19122, USA; 3 Center for Excellence in Genome Medicine and Research, King Abdulaziz University, Jeddah 21589, Saudi Arabia

**Keywords:** biogeography, cancer, dispersal, metastasis, migration paths, tumor

## Abstract

Understanding tumor progression and metastatic potential are important in cancer biology. Metastasis is the migration and colonization of clones in secondary tissues. Here, we posit that clone migration events between tumors resemble the dispersal of individuals between distinct geographic regions. This similarity makes Bayesian biogeographic analysis suitable for inferring cancer cell migration paths. We evaluated the accuracy of a Bayesian biogeography method (BBM) in inferring metastatic patterns and compared it with the accuracy of a parsimony-based approach (metastatic and clonal history integrative analysis, MACHINA) that has been specifically developed to infer clone migration patterns among tumors. We used computer-simulated datasets in which simple to complex migration patterns were modeled. BBM and MACHINA were effective in reliably reconstructing simple migration patterns from primary tumors to metastases. However, both of them exhibited a limited ability to accurately infer complex migration paths that involve the migration of clones from one metastatic tumor to another and from metastasis to the primary tumor. Therefore, advanced computational methods are still needed for the biologically realistic tracing of migration paths and to assess the relative preponderance of different types of seeding and reseeding events during cancer progression in patients.

## 1. Introduction

Cancer’s uniqueness emerges from its fundamental traits (hallmarks) of tumor growth, cell expansion, and dissemination from a tumor of origin (primary) to surrounding and distant tissues (metastases) [1]. Cancer cells gain the ability to migrate, invade, and modulate tumor microenvironments. Metastasizing clones may also trigger cellular plasticity and eventually colonize secondary tissues [2]. Due to genomic instability [3,4,5,6] and the generation of intratumor genetic heterogeneity, metastatic lesions cause failures of therapeutic approaches to eradicate metastases, subsequently making metastasis the dominant cause of cancer mortality [7,8]. A comprehensive understanding of the metastatic processes is essential for cancer biology, including cancer prognosis [9] and response to treatment [10].

Metastasis involves the migration of clones (i.e., cancer cells with identical genotypes) between primary and metastatic tumor sites, both of which accumulate somatic mutations over a patient’s life [11]. Thus, clones that originated from primary and metastatic tumors are evolutionarily related to each other, and their evolutionary relationship is depicted in a phylogeny. Moreover, tumor clone seeding or migration events are generally visualized in the form of migration graphs that show the relationship between the source and recipient-tumor site(s). These migration graphs can be constructed by using clone and tumor phylogenies that are frequently inferred from bulk sequencing and single-cell sequencing data [12,13,14,15,16].

In the past, clone seeding events were inferred by careful manual examination of cell and tumor phylogenies. However, as clone phylogenies are becoming larger due to sampling intensity (single-cell sequence data, collection of more metastases within a patient), it is expected that clone migration paths will also become more complex [17,18,19,20].

**Figure 1:**
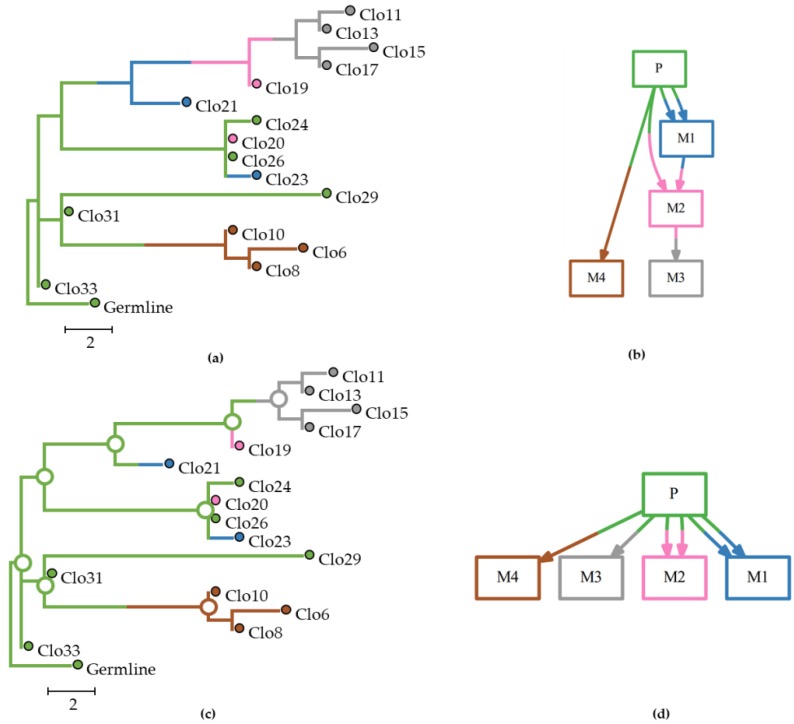
Analysis of an example dataset (m5Mseed76) with multisource seeding events (pM datasets) in which one tumor (M2) was seeded by clones from two other tumors (P and M1). This example dataset consisted of 16 clones and 52 characters from one primary and four metastatic tumors. Metastatic and clonal history integrative analysis (MACHINA) failed to infer the correct M1→M2 path. (**a**) The true (expected) clone phylogeny. (**b**) The true (expected) migration path. (**c**) Clone phylogeny inferred by MACHINA. (**d**) Migration graph predicted by MACHINA. Tumor clones and ancestral clones at the internal nodes are colored based on the source-tumor site: primary (green), and metastases M1 (blue), M2 (pink), M3 (gray), and M4 (brown).

Currently, there is only one computational approach for inferring migration events between tumors (metastatic and clonal history integrative analysis, MACHINA) [21]. For a given clone phylogeny, the MACHINA method first estimates the source-tumor site as the tumor of origin for the ancestral clones at the internal nodes. A migration event is inferred whenever the location of an ancestral node in the clone tree is different from its descendant node. MACHINA uses the maximum parsimony principle in which the optimality criteria (i.e., conditions to be reached for optimum migration inferences) include: (i) the number of migration events, i.e., an event where a clone migrates from one tumor to another, (ii) the number of comigration events, i.e., an event where two separate clones from a tumor site migrate to the same tumor (polyclonal seeding event); and (iii) the number of sources of the tumor site, i.e., sources of seeding clones [21]. This approach favors solutions that minimize the number of evolutionary and migration steps involved, which may underestimate the number of sources that contribute clones to metastases. The dataset of Figure 1 is one such example where the parsimony approach taken in MACHINA failed to infer clone seeding events between metastases, as it inferred the primary tumor as the only source of tumor cells for all seeding events. The BBM method performed better than MACHINA in this example dataset, as it produced the correct migration paths.

**Figure 2:**
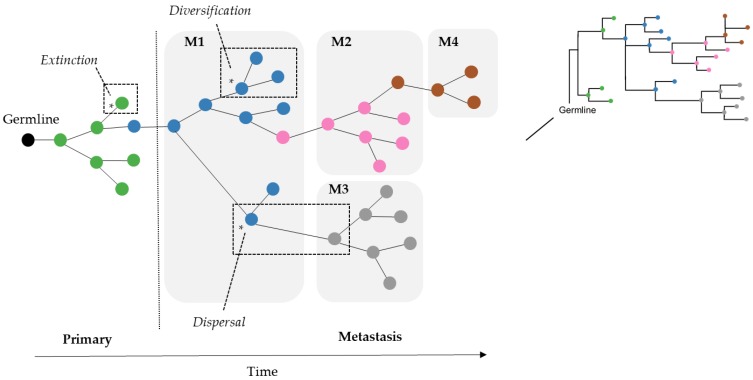
Cancer cells in tumors accumulate somatic mutations. Clones evolve over time within a tumor site and may go extinct or migrate to secondary tissues. In this figure, the example shows a tumor clone phylogeny with one primary and four metastatic tumor sites, each with multiple clones. Tumor migration events might be described by biogeographic processes (examples marked in dashed boxes) such as (i) dispersal: movements between areas, (ii) diversification: genetic divergence of lineages within an area, and (iii) extinction: disappearance of lineage(s) from an area. Tumor clones are colored based on the source of tumor site: primary (green), and metastases M1 (blue), M2 (pink), M3 (gray), and M4 (brown).

As a result, we wondered if the limitation of parsimony criteria could be overcome by the use of Bayesian approaches that do not use such counting principles. In the field of biogeography, many methods exist for inferring the origin and movement of species/populations between (geographic) areas. Some of these methods are expected to be well suited for understanding tumor migration paths because certain biogeographic processes are analogous to tumor clone seeding and colonization events, as seen in Figure 2. More specifically, clone seeding events between distinct tumor sites could be inferred by applying biogeographic methods that model events of species/populations movement between different areas (dispersal), genetic divergence of cell lineages within an area (diversification), and the disappearance of lineage(s) from an area (extinction), as shown in Figure 2 [22,23].

In the current study, we applied a Bayesian biogeographic approach that uses an evolutionary and spatial framework for inferring species migration routes to predict migration paths between tumor sites. Specifically, we tested the Bayesian Binary MCMC (BBM) method for inferring ancestral states [24] because this method uses a full hierarchical Bayesian approach to infer dispersal, diversification, and extinction events. We also compared BBM’s performance to that of MACHINA. We found that the performance of both methods is dictated by the number of tumor sites and the complexity of migration paths. Finally, we discuss the advantages of these methods and issues to be aware of when inferring tumor migration graphs.

## 2. Results

**Figure 3:**
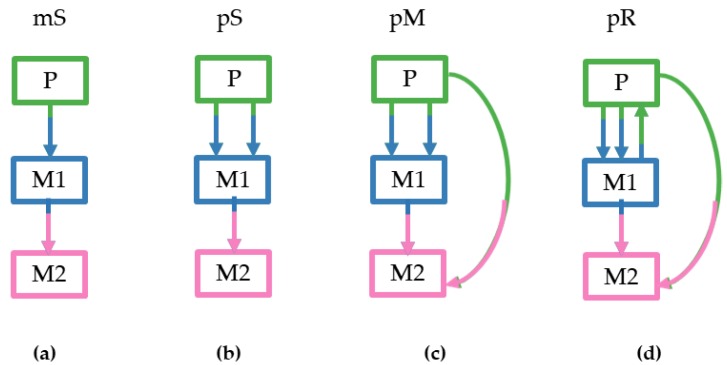
Examples of the four clone seeding scenarios as simulated by El-Kebir et al. [21], showing the direction of clone seeding events that were allowed to occur among tumor sites: (**a**) monoclonal single-source seeding (mS); (**b**) polyclonal single-source seeding (pS); (**c**) polyclonal multisource seeding (pM); (**d**) polyclonal reseeding (pR).

We considered four different tumor clone seeding scenarios based on the numbers of seeding clones (1–3), sources of the seeding clones (primary and/or metastatic), and the presence of reseeding of the primary tumors by clone(s) from metastases. For more details see the Methods section and Figure 3. These criteria dictated the pattern and complexity of simulated migration graphs that were different for all datasets. The monoclonal Single-source (mS) seeding was the simplest scenario followed by the polyclonal Single source seeding (pS), the polyclonal Multisource seeding (pM), and the polyclonal Reseeding (pR). Our test sample contained 20 simulated datasets for each type that were further subdivided into m5 and m8 datasets based on the number of tumor sites considered (5–7 and 8–11 tumors, respectively).

In our computational analyses, we used the Bayesian BBM method and the parsimony-based MACHINA algorithm (Parsimonious Migration History, PMH). We tested two different approaches based on the way that tree polytomies were treated in MACHINA: PMH-con, which does not attempt to resolve polytomies in the clone phylogeny, and PMH-TR, which does. More specifically, PMH-TR explores different tree topologies under which migration inferences are minimized, and jointly, refines any polytomies using the migration paths (for more details see the Methods section). For all analyzed methods, the source origin of the clone at the root of the phylogeny was assumed to be the primary tumor site. We evaluated these three approaches (BBM, PMH-con, and PMH-TR) to estimate their accuracy in inferring tumor migration paths.

### 2.1. Interpretation of BBM Results in the Context of Cancer

BBM infers ancestral distributions (locations) of species/populations at each node based on a given phylogeny and the distribution of species/populations in different locations. In cancer, clones constitute species/populations, and locations are the tumor (sampling) sites. BBM produces a source of origin of descendant clones at each ancestral node in the clone phylogeny, as well as the migration path with the highest posterior probability.

**Figure 4:**
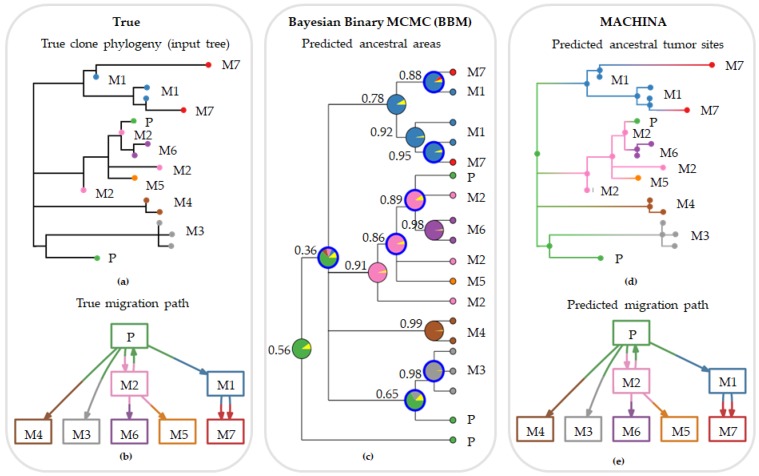
Inferred migration graphs for an example dataset (m8Rseed9; pR dataset). This dataset consisted of 18 clones and 66 characters from primary and seven metastatic tumors. (**a**) True (expected) clone phylogeny used as input tree for the migration inferences. (**b**) True migration path. (**c**) Ancestral areas predicted by Bayesian biogeography method (BBM) approach. Values next to the predicted ancestral ranges indicate Bayesian posterior probabilities. (**d**) Ancestral tumor sites predicted by PMH-con. PMH-TR produced the same result as PMH-con. (**e**) Predicted migration paths by MACHINA and BBM, which were consistent with the true migration graph. Tumor clones and ancestral clones at the internal nodes are colored based on the predicted source-tumor site: primary (green), and metastases M1 (blue), M2 (pink), M3 (gray), M4 (brown), M5 (orange), M6 (purple), and M7 (red). Unknown location of origin is marked as yellow in the pie charts of BBM.

Figure 4 shows a set of multiple and reseeding clone migrations, as seen in panel a, between primary and seven metastases, and the associated clone phylogeny, as seen in panel b. In panel c, we observe the predicted tumor sites at an internal node along with their posterior probabilities produced by BBM using the clone phylogeny in panel a. Corresponding results from MACHINA are shown in panel d. Both PMH-con and PMH-TR approaches produced the same result for this dataset. The predicted ancestral tumor sites (source-tumor sites at ancestral nodes) and migration paths were congruent with the expected (true) migration paths for this example dataset and all methods, as seen in panel e.

### 2.2. Impact of the Complexity of Migration Paths on the Inference Accuracy

**Figure 5:**
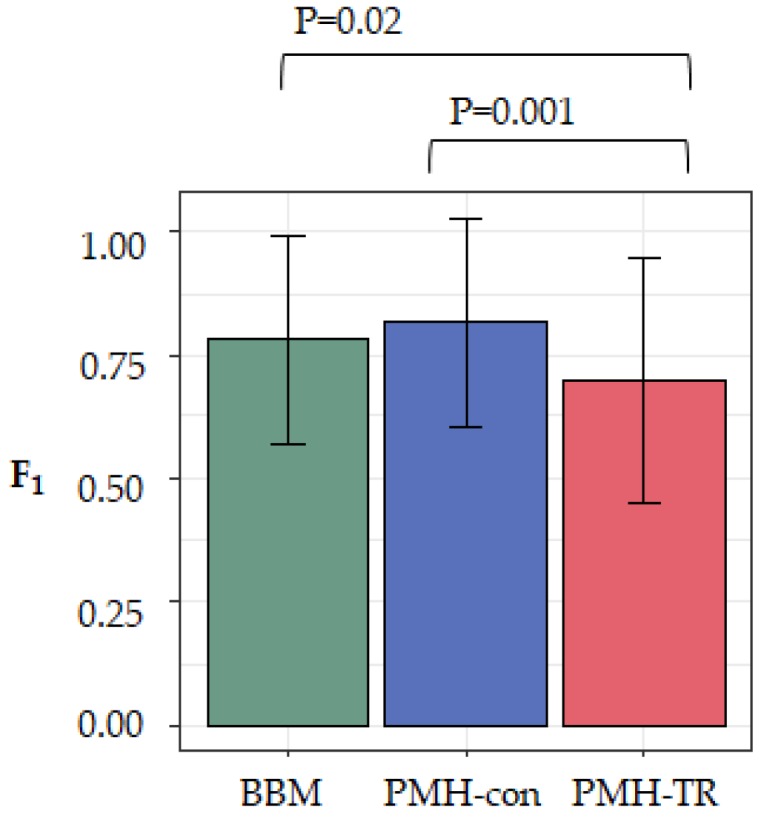
Overall performance based on F_1_-scores of the three evaluated approaches (BBM, PMH-con, and PMH-TR) on simulated datasets.

To evaluate the effect of the complexity of the dataset on the accuracy of the method, we computed F_1_-scores for each dataset (see Methods). Larger values of F_1_-scores indicate a more accurate inferred migration graph. We averaged the accuracy across all 20 datasets in each category and found that MACHINA and BBM to have similar overall performance, as seen in Figure 5. That is, there was not a significant accuracy difference between PMH-con and BBM (F_1_ = 0.82 and 0.79, respectively), but the performance of PMH-TR was consistently worse (F_1_ = 0.71), as seen in Figure 5. PMH-TR attempts to reduce the incorrect polyclonal seeding events inferred by PMH-con by jointly resolving polytomies and inferring migration paths. However, this feature introduces more errors in the migration inferences than PMH-con, most likely because PMH-TR often resolves polytomies incorrectly, as seen in Figure 5.

**Figure 6:**
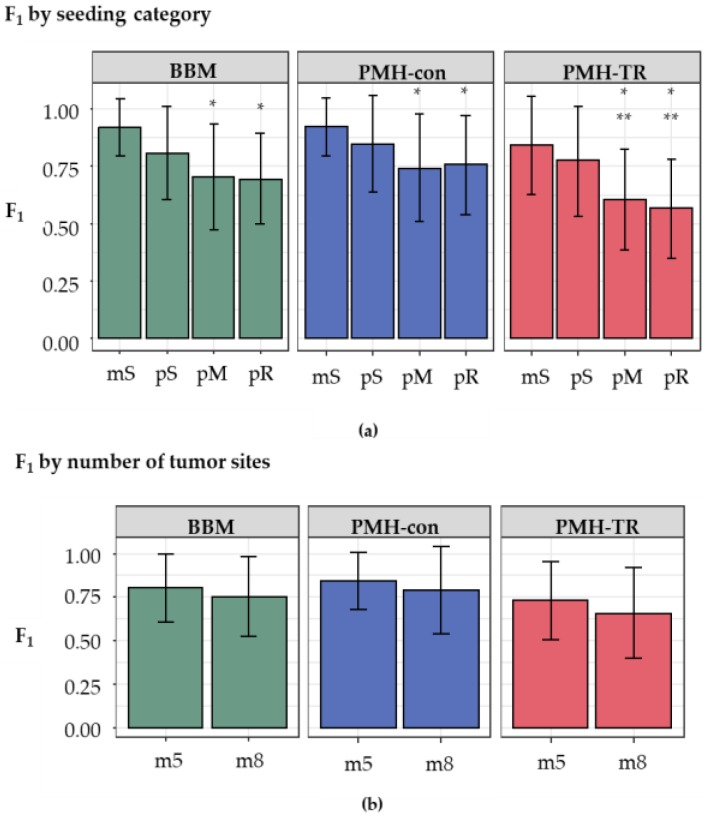
Overall performance (F_1_-score, which is the harmonic mean of precision and recall) of different approaches for (**a**) increasing complexity of migration paths and (**b**) different number of tumor sites. Complexity of the migration graph for different seeding types was in the order: mS < pS < pM < pR. The number of tumor sites were categorized as m5 and m8 datasets (5–7, and 8–11 tumors, respectively). Differences in values of F_1_-score on seeding category and tumor count were examined through *t*-test and are marked when significant (*: *p* < 0.05 with mS; **: *p* < 0.05 with pS).

We next examined the impact of the increasing complexity in the history of metastatic tumor evolution, i.e., the presence of polyclonal seeding events (pS), the presence of multiple source tumor sites for seeding a metastatic tumor (pM), and the presence of reseeding event (pR). The inference of correct metastatic patterns is clearly a function of the complexity of the migration graphs, as the accuracy decreases with increasing complexity, as seen in Figure 6a. Migration paths in datasets with monoclonal seeding events (mS) were the easiest to reconstruct correctly, with overall accuracy ranging from 0.84–0.92. In contrast, the presence of polyclonal reseeding (pR) made the inference of metastatic patterns very challenging, as the accuracy declined to 0.58–0.76.

Next, we conducted statistical tests to compare F_1_-scores across complexity classes, as seen in Table S1. The null hypothesis of equal effect between different (simple or complex) migration schemes on the accuracy was rejected for eight out of 18 pairs of seeding scenarios at *p* < 0.01. Interestingly, a comparison of the accuracy for mS with pM (BBM and PMH-con) and pR scenarios (BBM, PMH-con, and PMH-TR) showed no significant impact of the complexity. The difference of F_1_-scores between BBM and PMH-con was also not substantial in any clone seeding scenario, but PMH-TR always produced much lower F_1_-score than BBM and PMH-con. PMH-TR was also not robust to these complexities, and F_1_-scores decreased with increasing complexity. Thus, the inference of migration paths becomes hard for both BBM and MACHINA when the migration patterns are complex.

We also examined the impact of the number of tumor sites within a dataset. Datasets were grouped into two categories, those with a small number of tumors (5–7 tumors per dataset, m5 datasets) and those with a larger number of tumors (8–11 tumors per dataset, m8 datasets). Although the average F_1_-scores of m5 datasets were slightly higher than those of the m8 for all methods (0.82 and 0.76 for BBM; 0.85 and 0.79 for PMH-con; and 0.74 and 0.67 for PMH-TR, respectively), as seen in Figure 6b, these differences were not statistically significant in the *t*-tests.

### 2.3. Accuracy for Different Types of Migration Paths

**Figure 7:**
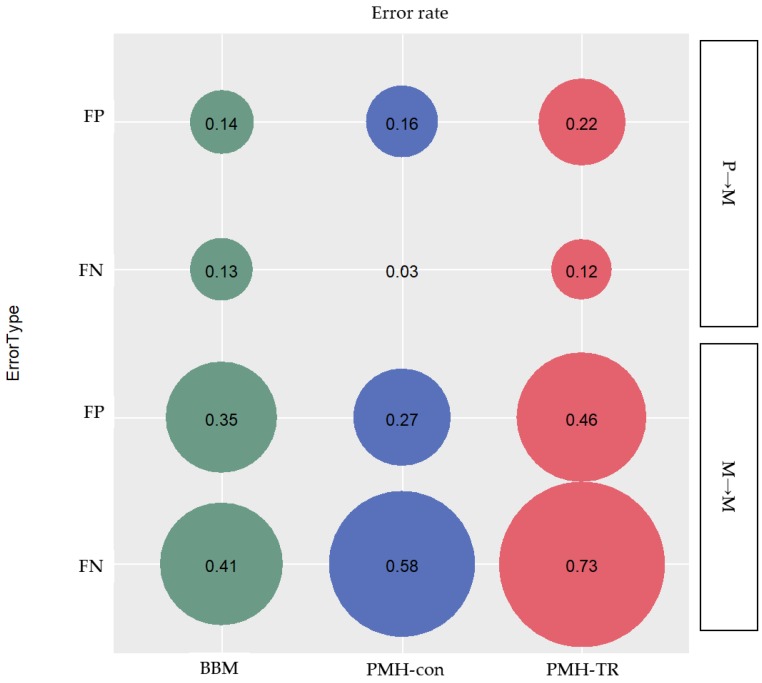
Error rate of incorrectly (false positives; FPs), and not identified (false negatives; FNs) inferred migration paths for the two categories of clone seeding events between different tumor sites: (i) primary and metastatic site(s) (P→M), and (ii) metastatic sites (M→M).

Migration paths can be classified into three categories based on the type of clone seeding from (i) primary to metastatic tumor site(s) (P→M), (ii) metastatic to another metastatic tumor site (M→M), and (iii) metastatic to primary tumor site (M→P). We assessed the proportion of inferred paths that were incorrect (false positives; FPs) and the proportion of correct paths that were not identified (false negatives; FNs), as seen in Figure 7.

#### 2.3.1. Accuracy of Migration Paths from Primary to the Metastatic Tumor Site (P→M Path)

We found that PMH-con outperformed other methods in identifying correct P→M paths. The average error rate of FNs of P→M paths was only 3%, while the errors inferred by BBM and PMH-TR were 13% and 12%, respectively, as seen in Figure 7. However, PMH-con produced more incorrect P→M paths than BBM with error rates of 16% and 14% for PMH-con and BBM, respectively. PMH-TR produced a much larger number of incorrect P→M paths than other methods, with an error rate of incorrect paths equal to 22%. That being said, the PMH-con method might have recovered more correct P→M paths than BBM, but the collection of paths inferred included many incorrect P→M paths. This pattern of performance of MACHINA may be explained by the fact that MACHINA minimizes the number of source-tumor sites in the inference of migration graphs. Consequently, MACHINA tends to generate migration schemes with a minimum number of source sites for a given dataset, e.g., one-step P→M paths will be favored over multistep P→M→M paths.

#### 2.3.2. Accuracy of Migration Paths from Metastatic to another Metastatic Tumor Site (M→M Path)

We found that PMH-con outperformed the other methods in identifying correct P→M paths. The average error rate of FNs of P→M paths was only 3%, while the errors inferred by BBM and PMH-TR were 13% and 12%, respectively, as seen in Figure 7. However, PMH-con produced more incorrect P→M paths than BBM with error rates of 16% and 14% for PMH-con and BBM, respectively. PMH-TR produced a much larger number of incorrect P→M paths than the other methods, with an error rate of incorrect paths equal to 22%. That being said the PMH-con method might have recovered more correct P→M paths than BBM, but these include incorrect P→M paths.

#### 2.3.3. Accuracy of Migration Paths from Metastatic to the Primary Tumor Site (M→P Path)

Similar to the inference of M→M paths, the ability to detect M→P paths was low for all of the methods (pR datasets). BBM and PMH-con produced only half of these M→P paths. Among the methods, PMH-TR performed the worst, as it rarely identified M→P paths.

Nevertheless, MACHINA did not produce any more incorrect M→P paths than BBM, most likely because MACHINA minimizes the number of source tumor sites and restricts producing migration paths that start from metastatic tumor sites. Consequently, inferences of incorrect M→P paths can be prevented more efficiently while using MACHINA than BBM. However, it is important to note that this restriction in the MACHINA method produces, more frequently, incorrect inferences of P→M paths, and less frequently, correct inferences of M→M paths, compared to BBM.

#### 2.3.4. Overall Accuracy of Migration Paths

Overall, the BBM and MACHINA methods can detect only P→M accurately. We found that 26 and 30 inferred migration graphs among 80 datasets by BBM and PMH-con, respectively, were entirely correct, i.e., they were identical to their correct migration graphs. Around half of these datasets (11 and 15 datasets, respectively) did not contain any M→M or M→P paths. We need to be aware of this error pattern when these methods are used for empirical data analysis.

Moreover, BBM is easy to run as it is part of the RASP toolkit, but runs required ~1.5 hours, on average, and depended on the number of areas considered for the analysis (1–3.5 hours). BBM does not produce a plot of migration paths, and so, at present, it has to be drawn manually. MACHINA software is less user-friendly than BBM, as we found it to be challenging to install. It runs very fast (only a few seconds). MACHINA produces a plot of migration paths, which can be easily visualized by using software such as Graphviz (some online versions are also available, e.g., http://www.webgraphviz.com/).

## 3. Discussion

In this study, we evaluated a Bayesian biogeographic method as a potential alternative approach for inferring accurate cancer cell migration events between tumor sites (tumor biogeography). Although a method in biogeography has been applied to this purpose in the past [23], the accuracy of biogeographic methods is being explored for the first time in this study. Here, we applied BBM [24], because it uses a phylogenetic tree and infers dispersal patterns. We also tested the accuracy of the MACHINA method for inferring migration paths in metastasis.

Overall, we found that BBM and MACHINA produced similar results, with high accuracy in predicting a large number of migration paths correctly as long as the clone migration patterns were simple. More specifically, the complexity of datasets, i.e., presence of polyclonal seeding tumors (single source polyclonal, e.g., P→M1 and P→M1, or multiple source seeding, e.g., P→M1→M2 and P→M2) and reseeding events (M→P), impinged on the performance of the evaluated methods. On the other hand, single clone seeding events from a primary to metastasis (P→M) or between metastases (M→M) were less problematic to infer.

In more complex migration patterns between tumors, such as those with multiple source seeding in which a tumor is seeded from more than one tumor, e.g., P→M1→M2 and P→M2, the accuracy of both methods became low. The number of tumor sites and the complexity of migration graphs strongly impacted the performance of both methods. We also found that the performance of BBM excels in inferring migration paths between metastases (M→M), while MACHINA performs better for paths between primary to metastasis (P→M), and vice versa (M→P).

Each inferred migration graph generally contained one or a few incorrect migration paths. We found that most of P→M paths can be identified, but incorrect P→M paths will be additionally produced. Inferences of M→M and M→P paths are hard to identify correctly. This result could be explained by the constraint of the primary tumor as the starting tumor site for migration inferences in both MACHINA and BBM. The latter finding might indicate that the two methods are complementary to one another in validating and improving the accuracy of the metastatic inferences.

Even though BBM did not perform better than MACHINA in our direct comparisons, it is likely to be more useful because it provides more detailed information about migration paths than MACHINA. Being a Bayesian approach, BBM assigns posterior probabilities to ancestral range states for nodes in the clone phylogeny, and thus, relative probabilities of different biogeographical (dispersal, diversification, and extinction) events (if any) are produced for each ancestral node. An example is shown in Figure 4, in which the dataset included a large number of tumors, migration of multiple clones from one tumor to another, and reseeding events. In this case, the migration inferences from MACHINA and BBM were congruent with the true migration path. MACHINA produced only one possible ancestor tumor site, as seen in Figure 4d, but BBM assigns one location (or more) as a probable origin for the lineage and suggests the migration path (including probable dispersal, diversification and extinction events) for the ancestor node along with the probability for the location(s) of origin assigned at the node (for more details, see Methods section). Figure S1 shows the prediction of BBM migration paths as a result of dispersal events. For example, for the node with the assigned migration path M1→M1M7→M1ĤM7 on the top of the phylogenetic tree of the Figure S1, we observe that the tumor M1 is suggested as the source of origin for the lineage. BBM further indicates that a clone from M1 migrated to tumor M7 through dispersal (event marked as blue circle around the ancestral node), where clones eventually diverged.

Examining more thoroughly the inferred migration paths in Figure S1, we also observe diversification events, denoted as, e.g., M2^M2. In cancer, the spread of clones is modelled through seed composition (number of clones that migrated), seed source (tumors participating in metastatic cascades), and timing (when clones diverged) [17]. BBM essentially delineates the location where genetic divergences of clones have occurred, indicating the series of tumor genetic divergence. The information on biogeographic processes inferred by BBM could be used to describe tumor clone evolutionary events in more detail. We argue that BBM is a potentially good method for modeling metastatic progression. The patterns produced by BBM may be used as a scaffold to begin to generate information on the source tumor for metastases, the number of clones involved in the initial formation of metastasis, and the route of metastatic clones among tumors in a cancer patient. This knowledge will ultimately inform the relative contribution of mutation and migration in causing intratumor heterogeneity, which is the leading cause of treatment failure and cancer mortality.

Ultimately, we must conclude that the methods available for tumor biogeography [21,23] are indeed in their infancy, as the overall accuracies are rather modest for both MACHINA and BBM. In spite of these limitations, our results clearly show promise for the application of computational methods in biogeography to infer migration graphs in cancer. By uniting the fields of population and species biogeography with tumor biogeography, we hope that it will be possible to accelerate the progress in developing more accurate computational methods for inferring migration paths between metastatic tumors. These developments will likely come from advancing the statistical framework of contemporary biogeographic methods that can integrate phylogenetic, longitudinal, and spatial signals in sequence variation. Progressively higher resolution data capturing finer details of spatiotemporal heterogeneity and evolution of tumors is likely to aid in the reliable reconstruction of migration patterns.

## 4. Materials and Methods

### 4.1. Simulated Datasets and Seeding Scenarios

To ensure a direct comparison, we used the same simulated datasets as those used to evaluate MACHINA [21]. In brief, these datasets were simulated based on the model of clone evolution and tumor growth with cell migration events modeled based on ref. [25]. Tumor migration schemes were designed and simulated based on migration (seeding) events occurring after cell cycle events (replication and death). The probabilities of clone seeding events were proportional to the number of cells in a tumor site and of the drivers considered therein. Tumor cells were allowed to migrate from an existing to another anatomical site (tumor site).

Based on the number of seeding clones (single cell, monoclonal; and group of cells, polyclonal), the number of source sites of seeding clones (single source site and multiple source site), and the presence of reseeding events (from metastatic to primary), datasets were classified into four categories. The first category of simulated tumor migration schemes included cells from one clone that could migrate from a tumor site (primary or metastatic) to another (single-source monoclonal seeding events, mS). In the second category, at least one clone per tumor site seeded another tumor site (single-source polyclonal seeding events, pS). In the third category, at least one clone per tumor site seeded two different tumor sites (multisource seeding events, pM). The fourth category contained reseeding events from metastasis to primary site, together with monoclonal seeding, single-source polyclonal seeding, and/or multisource polyclonal seeding events (reseeding, pR), as seen in Figure 3.

All the datasets were further subdivided into m5 and m8 datasets based on the number of tumor sites considered (5–7 [7] and 8–11 tumors, respectively). In total, we obtained 80 simulated datasets of clone sequences and clonal composition of primary and metastatic tumor sites, together with their histories of clone seeding events from ref. [21]. The number of primary and metastatic tumor sites ranged from 5–11. Datasets included 7–28 clones, and the number of single nucleotide variants (SNVs) was 9–99, as seen in Table S2**,** [21]. All clone phylogenies and the information of tumor sites that contain clones are available at https://github.com/raphael-group/machina/tree/master/data.

### 4.2. Methods for Migration Events Inference

To infer migration paths, we used the MACHINA and BBM methods. Because our interest was in testing the ability of computational methods to infer migration paths, we provided the true clone phylogenies and tumor sites. In the input tree phylogenies, branch lengths are the number of mutations, because such phylogenies are generally used on cancer research [7,26,27,28,29]. The computer-simulated data were in a binary (i.e., two-state) character system, where there were two character states for nucleotides corresponding to the normal (germline) base and to the base substitution. Mutations in the simulated sequence arose only once, meaning that the substitution process happens only towards one direction with no backward or parallel substitution changes at each nucleotide position (no homoplasy). This substitution process is in accordance with the clonal evolution model which proposes that tumors arise from a single mutated cell accumulating additional mutations [30]. When sequence data have no homoplasy, then maximum parsimony is expected to produce the correct phylogeny [31]. For that reason, we used maximum parsimony (MP) method in MEGA-CC [32] to reconstruct correct phylogenies from simulated clone sequences. All phylogenies were rooted by normal (germline) cells without any somatic mutations.

#### 4.2.1. MACHINA Method

The Parsimonious Migration History (MACHINA) approach uses (joint) inferences of tumor clone phylogenies and/or metastatic migration histories by using DNA sequencing data [21]. We are neither examining nor discussing further the part related to clone phylogenies inferred by MACHINA, as it is beyond the scope of this study and has been discussed elsewhere [33]. We focus on the inference of migration path given the correct clone history. The MACHINA method predicts and assigns a location for each ancestral clone by using a provided list of connections between nodes and locations of tip nodes.

For MACHINA tumor migration inferences, we performed two types of analyses. In the first analysis, we constrained the ancestor at the root of the tree to be a primary tumor, and in the second, we used the Sankoff algorithm, which allowed any tumor site to be the ancestor at the root. We found that the second option produced many results, as many as 800 solutions for some datasets, as seen in Figure S2; thus, we primarily discussed findings from the constrained analysis. We also used the Parsimonious Migration History with Tree Resolution (PMH-TR) with primary tumor site constrained to examine the effect of tree polytomies in the migration inferences (analyses named as *PMH-TR*). PMH-TR resolves polytomies of a given clone phylogeny by searching for topologies that reduce the number of migration events and, subsequently, refining polytomies in a given clone tree using the migration pattern). For each approach, we allowed all possible seeding scenarios (monoclonal seeding, single/multisource polyclonal seeding, and reseeding events) to be explored. No upper bounds were placed on the number of migrations and comigrations.

#### 4.2.2. BBM Method

To infer tumor migration patterns, we used the biogeographic Bayesian Binary MCMC (BBM) method for ancestral state [24]. BBM follows analogous transition state changes among (discrete) areas similar to nucleotide changes during DNA substitution models [34,35]. Several biogeographic methods are developed to infer migration events of species and populations with each method treating the evolutionary (cladogenesis and anagenesis events) and biogeographic (dispersal, diversification, extinction, vicariance) processes differently (for a thorough review, see ref [22]). Here, we have used a Bayesian approach, BBM, because it integrates different types of information such as phylogenetic signal in terms of posterior probabilities and/or topology (which yield Bayesian posterior probabilities for ancestor’s ranges), longitudinal (divergence times), and geological/spatial (in the form of geographical coordinates or unit areas). In the current analysis, we provided the true clone phylogeny as evolutionary-type information, together with true tumor sites’ locations as spatial information. This way, the same inputs were given to BBM and MACHINA.

We employed BBM analyses in RASP v4 [36]. The Markov-chain Monte Carlo chains were run three times for 5,000,000 generations to assure that MCMC chains would reach stationarity and convergence. The stationary rate frequencies and reconstructed states were sampled every 1000 generations. The first 1000 states (burn-in) were eliminated. The three independent runs were combined into a single result, which contained the inferred ancestor ranges and their probabilities. BBM ancestor ranges were estimated under the fixed Jukes–Cantor (JC69) [37] with equal dispersal rates, i.e., equal seeding rates among clones. In particular, we considered that clone seeding events between tumor sites are plausible in a migration scenario in which (a) size of the tumor site is considered not to affect dispersal patterns; (b) carrying capacities of areas and dispersal rates between tumor sites are equal; and (c) directionality of dispersal routes is allowed from any location to another [35]. The number of areas in ancestral nodes was constrained based on the number of tumor sites simulated for each dataset: (A) P, (B) M1, (C) M2, (D) M3, (E) M4, (F) M5, (G) M6, (H) M7, (I) M8, (J) M9, and (K) M10, where P, stands for primary tumor, and Mn for metastatic tumor site with *n* = 1–10.

The BBM method does not have the option to constrain a specific area as the ancestor at the root of the tree. To induce this constraint, we used clone phylogenies with the germline sequence as an outgroup, and we assigned the primary tumor site to be the location of the outgroup (analyses named as *BBM*).

### 4.3. Performance Measures

We evaluated the performances of the MACHINA and BBM methods by comparing the inferred G and simulated (expected) G* migration graphs, which were composed of migration paths, e.g., Figure 1b,d. We counted the number of correctly inferred migration paths (true positives; TPs), those not identified (false negative; FNs), and incorrect paths (false positives; FPs). We then computed F1-score (1) for each dataset, which is the harmonic mean of precision (2) and recall (3):

(1) $$F_{1}\;=\;2\text{ × }\frac{\mathrm{precisi}(1)\mathrm{on}\text{ × }\mathrm{recall}}{\mathrm{precision}\;+\;\mathrm{recall}}$$

where

(2) $$\mathrm{precision}(G,\;G^{*})\;=\;\frac{\mathrm{TP}}{\mathrm{TP}\;+\;\mathrm{FP}}$$

and

(3) $$\mathrm{recall}(G,\;G^{*})\;=\;\frac{\mathrm{TP}}{\mathrm{TP}\;+\;\mathrm{FN}}$$

We conducted Z-tests to assess the significance of the difference in F_1_-scores between seeding scenarios and for each approach.

## 5. Conclusions

Currently, there is only one computational method in cancer research that infers metastatic histories. Here, we introduce a ‘tumor biogeography’ approach for delineating clone migration events between tumors. We have shown that overall biogeographic methods perform equally as well as MACHINA in inferring migration patterns in metastasis. Both methods produce accurate migration inferences for datasets with simple migration graphs. The performance of both methods was impacted by the complexity of datasets in terms of the number of tumor sites, presence of polyclonal seeding tumors, or multiple source seeding and reseeding events.

In conclusion, we endorse the use of biogeographic methods for inferring metastatic origin and routes as a sophisticated alternative framework. Biogeographic methods can integrate phylogenetic, longitudinal, and spatial signals of sequence data, while the current method for migration inferences between tumors cannot accommodate cancer heterogeneity in such a continuum. Progressively, more sequence data, featuring the great evolutionary and spatiotemporal heterogeneity of tumors, are becoming available. A cohort of high-accuracy that would bridge this three-dimension scale will be of high value in determining treatment strategies, appropriately designed to target the heterogeneity of metastases.

## Supplementary Materials

The following are available online at https://www.mdpi.com/2072-6694/11/12/1880/s1, Figure S1: Inferred migration graph by BBM for the example dataset of Figure 4. Here the inferred migration paths due to dispersal events (blue circles around the nodes) are shown, e.g., M1→M1M7→M1│M7. Values next to the predicted ancestral ranges indicate Bayesian posterior probabilities, Figure S2: Parsimonious Migration History (PMH) under the unconstrained mode. Inferred number of migration graphs ranged from 1–800 with the majority of the datasets to have less than 50 inferred migration graphs per input, Table S1: Comparison of the overall performances (F1-scores) between the four seeding scenarios for each analyzed approach. The z-scores are shown below the diagonal, and the corresponding P values are shown above the diagonal, Table S2: Datasets information regarding the number of anatomical (tumor) sites, clones, and SNVs included. Datasets in which a clone from a metastatic tumor site was found at the root at the rest of datasets are marked in boldface.
