# Supplementary material for: Delineation of Tumor Migration Paths by Using a Bayesian Biogeographic Approach
Source: Cancers (Basel). 2019 Nov 27;11(12):1880. doi: 10.3390/cancers11121880 (PMC6966534; doi:10.3390/cancers11121880)
Supplement: Supplementary file 1 [file cancers-11-01880-s001.zip › cancers-632025-SI/Chroni_et_al_Supplementary/Supplementary Table S1.pdf]

Supplemental Table S1. Comparison of the overall performances (F_1-scores) between the four seeding scenarios for each analyzed approach. The z-scores are shown below the diagonal, and the corresponding P values are shown above the diagonal.

**BBM**				
	mS	pS	pM	pR
mS		**0.06**	0.00	0.00
pS	1.93		**0.14**	**0.11**
pM	3.49	1.45		**0.40**
pR	4.04	1.61	0.00	
**PMH-con**				
mS		**0.18**	0.00	0.01
pS	1.26		**0.12**	**0.16**
pM	2.98	1.55		**0.38**
pR	2.84	1.34	-0.27	
**PMH-TR**				
mS		**0.27**	0.00	0.00
pS	0.87		0.03	0.01
pM	3.26	2.24		**0.34**
pR	3.82	2.77	0.57	

mS, monoclonal single-source seeding; pS, polyclonal single-source seeding; pM, polyclonal multi-source seeding; and pR, polyclonal reseeding
