# Supplementary material for: Delineation of Tumor Migration Paths by Using a Bayesian Biogeographic Approach
Source: Cancers (Basel). 2019 Nov 27;11(12):1880. doi: 10.3390/cancers11121880 (PMC6966534; doi:10.3390/cancers11121880)
Supplement: Supplementary file 1 [file cancers-11-01880-s001.zip › cancers-632025-SI/Chroni_et_al_Supplementary/Supplementary Table S2.pdf]

Supplemental Table S2. Datasets information regarding the number of anatomical (tumor) sites, clones, and SNVs included. Datasets in which a clone from a metastatic tumor site was found at the root at the rest of datasets are marked in boldface.

Dataset_ID	Anatomical_sites	Clones	SNVs
m5Mseed694	5	14	41
m8Rseed1070	8	18	81
m5Sseed17	5	11	44
m8mSseed10	8	18	43
m5mSseed9	5	7	9
m5Mseed865	5	10	34
m8mSseed2	9	14	27
m8Rseed394	9	20	38
**m8Mseed241**	8	20	67
**m8Mseed45**	8	19	93
m8Sseed2	8	20	64
**m5Sseed25**	5	12	26
m8mSseed7	8	20	50
m5Mseed565	5	12	35
m8Rseed383	8	25	83
m8mSseed3	9	24	61
m5Rseed17	6	15	53
m5mSseed5	5	13	36
m8Sseed54	8	19	70
**m5Mseed473**	7	19	73
**m5mSseed0**	5	16	35
m8Rseed30342	8	18	50
m8mSseed8	9	24	78
**m5Sseed81**	5	16	32
m5mSseed2	5	10	16
m8Sseed5	8	24	67
m5Sseed32	5	11	46
m5Mseed512	5	14	47
m5mSseed10	5	12	22
**m5Sseed23**	5	13	50
m5mSseed12	5	11	19
m8Rseed905	8	18	54
m5Rseed538	5	15	55
**m8Mseed35**	9	21	65
m5Sseed49	5	12	27
m8Mseed7	8	22	66
m8Sseed0	10	24	65
m8Rseed157	11	28	68
m5Rseed247	5	15	42

m5Mseed76	5	16	52
m5mSseed3	6	22	53
m5mSseed8	5	15	53
**m8mSseed0**	9	23	50
m5Mseed907	6	14	73
m8Rseed981	9	20	99
**m8Rseed9**	8	18	66
**m8Mseed243**	9	23	74
m8Sseed69	8	17	64
m5Rseed571	6	14	36
m8Sseed31	8	18	50
m5Sseed40	6	16	46
m5Rseed955	5	13	32
m8Mseed172	10	26	64
m5Mseed534	5	12	33
m5Rseed950	7	15	38
m8mSseed5	8	18	52
m5Sseed62	5	14	31
**m8Mseed239**	8	17	65
m8Rseed10157	8	25	47
m5Mseed209	5	14	51
m5Rseed981	5	16	61
m8Sseed35	8	24	75
m5Sseed31	5	14	35
m8Mseed76	9	19	77
m5mSseed7	5	13	35
m8mSseed12	9	17	48
m5mSseed4	5	12	33
m5Sseed35	5	15	41
m8Mseed216	8	21	58
m8mSseed4	8	19	60
m8Mseed19	8	18	54
**m5Rseed2155**	7	16	48
m5Rseed1140	5	12	19
m8Sseed12	8	21	36
m5Rseed518	5	13	52
m5Mseed545	6	16	46
**m8Rseed10046**	8	21	75
**m8mSseed9**	9	18	32
m8Sseed37	8	19	71
