# Supplementary figures and images for: Delineation of Tumor Migration Paths by Using a Bayesian Biogeographic Approach
Source: Cancers (Basel). 2019 Nov 27;11(12):1880. doi: 10.3390/cancers11121880 (PMC6966534; doi:10.3390/cancers11121880)

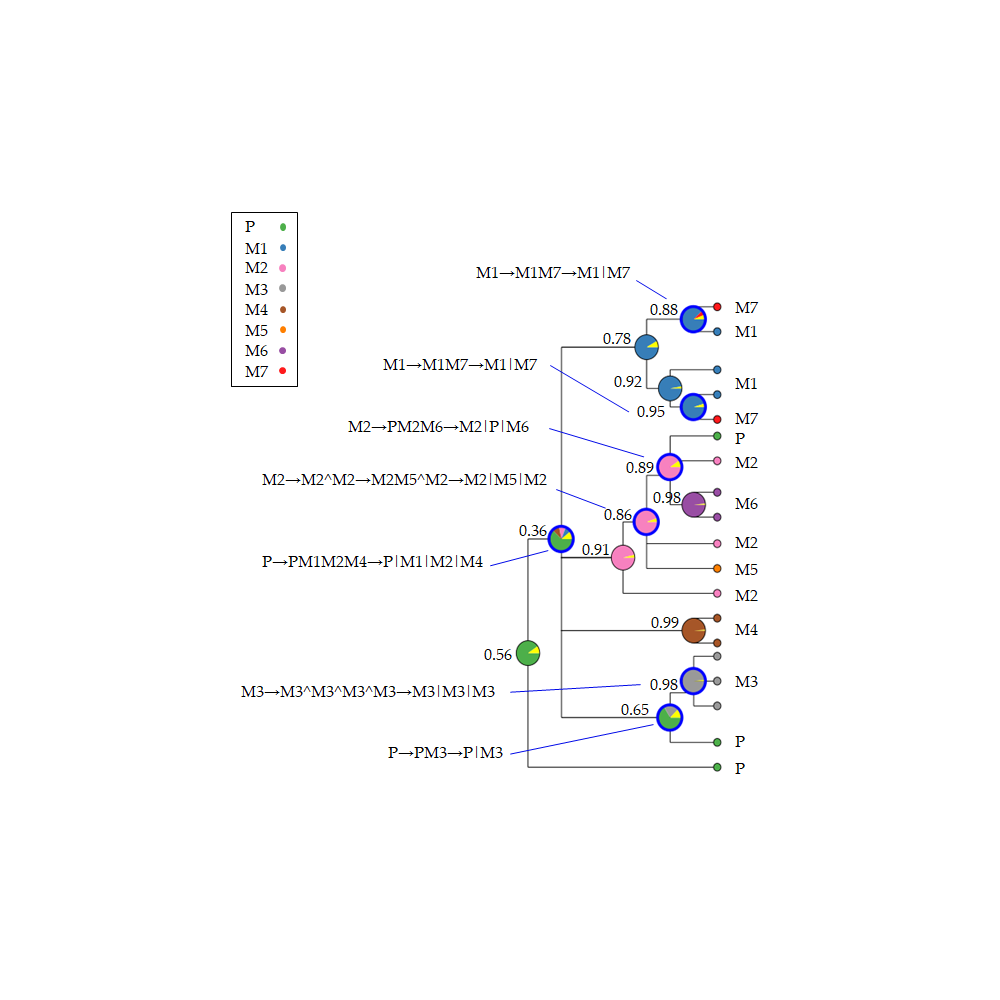

Supplement: Supplementary file 1 [file cancers-11-01880-s001.zip › cancers-632025-SI/Chroni_et_al_Supplementary/FigureS1.tif]

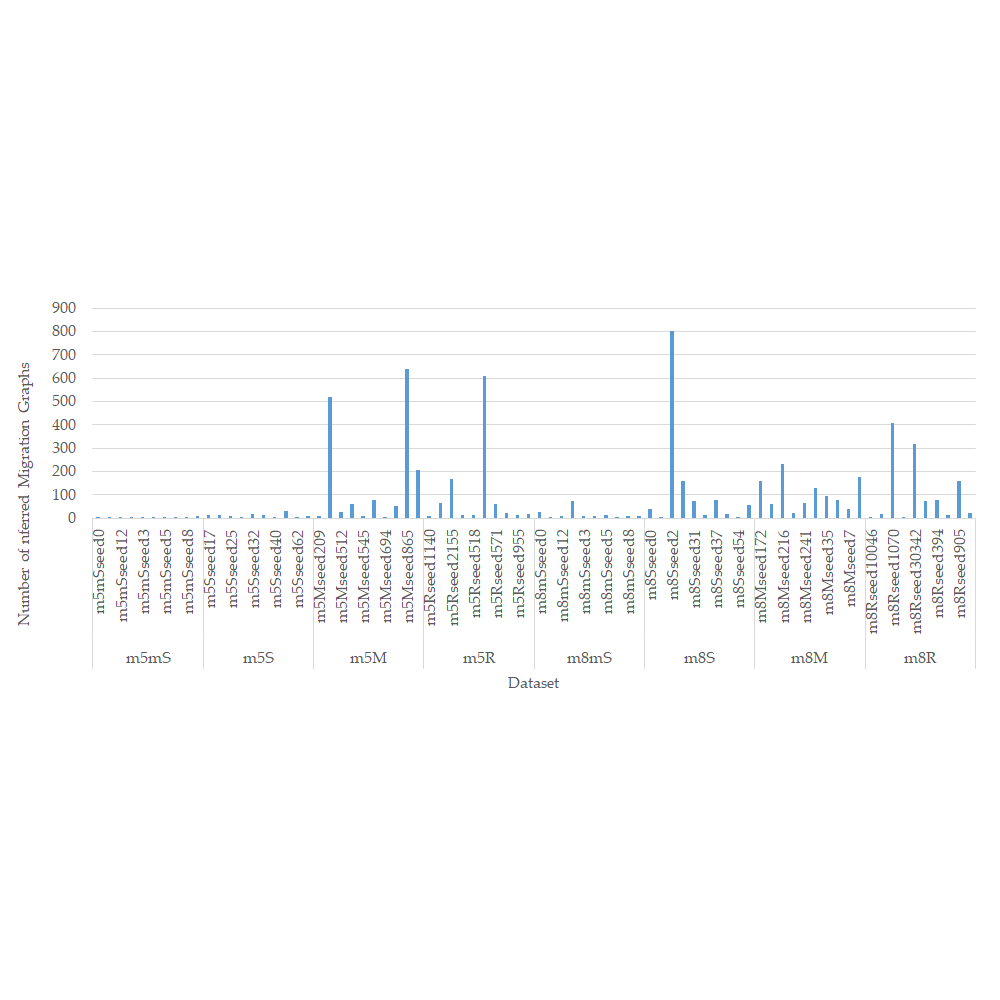

Supplement: Supplementary file 1 [file cancers-11-01880-s001.zip › cancers-632025-SI/Chroni_et_al_Supplementary/FigureS2.tif]
